# Validation of the vignette-based German Exercise Causality Orientation Scale (G-ECOS)

**DOI:** 10.1371/journal.pone.0223643

**Published:** 2019-10-10

**Authors:** Lena Busch, Till Utesch, Bernd Strauss

**Affiliations:** Department of Sport Psychology, Institute of Sport and Exercise Sciences, University of Münster, Horstmarer Landweg, Münster, Germany; Universiti Sains Malaysia, MALAYSIA

## Abstract

The Self-Determination Theory has been applied to explain behaviour in numerous contexts and cultures. In the exercise context, causality orientations (autonomy, control, impersonal) are important to describe individual differences in initiation and maintenance of health behaviour. The assessment of exercise causality orientations can be a key element to improve predictions of motivated health and exercise behaviour. Nevertheless, a scale to measure exercise causality orientations has not been established in German yet. Thus, it was the aim of the present work to translate the Exercise Causality Orientations Scale to German and to test it throughout three studies. The German G-ECOS questionnaire was cross-validated via confirmatory factor analyses in two separate samples. Both Study 1 (*n* = 306, 72.60% female, age *M* = 26.00, *SD* = 5.66; CFI = .96) and Study 2 (*n* = 320, 70.94% female, age *M* = 29.00, *SD* = 3.54; CFI = .95) indicated a good model fits. In a further Study 3 (*n* = 548, 62.50% female, age *M* = 30.17, *SD* = 11.91), the relations between exercise causality orientations and other SDT related constructs were examined. The correlations indicated positive associations between autonomy causality orientation and intrinsic regulation, intrinsic exercise participation goals, and exercise basic needs satisfaction. Overall, the assessment of exercise causality orientations can be useful in analysing and potentially predicting motivated exercise behaviour.

## Introduction

Physical activity has been shown to be an important predictor of health status, preventing chronic physical and mental diseases such as type-II diabetes, hypertension, and depression [1,2]. Therefore, it has become an aim of health care providers, psychologists, and sport scientists to enhance physical health and physical activity in men and women [3,4]. In this context, it is important to identify and to explain the underlying reasons and mechanisms that are associated with exercise behavior. One widely used approach that can serve to explain motivated behaviour (e.g., health behaviour) is the Self-Determination Theory [5,6]. The Self-Determination Theory (SDT) consists of several complimentary sub-theories and has successfully been applied across cultures. To date, its application to the health and exercise context has been reviewed and supported by numerous studies [7,8]. Thus, to gain a comprehensive understanding of the underlying processes of motivated behavior, it is important to provide a complete set of assessment tools measuring motivational aspects associated with the SDT. Furthermore, to provide valuable international and cross-cultural research, it is crucial to translate and validate such assessment tools into other languages.

### Self determination theory

**Fig 1:**
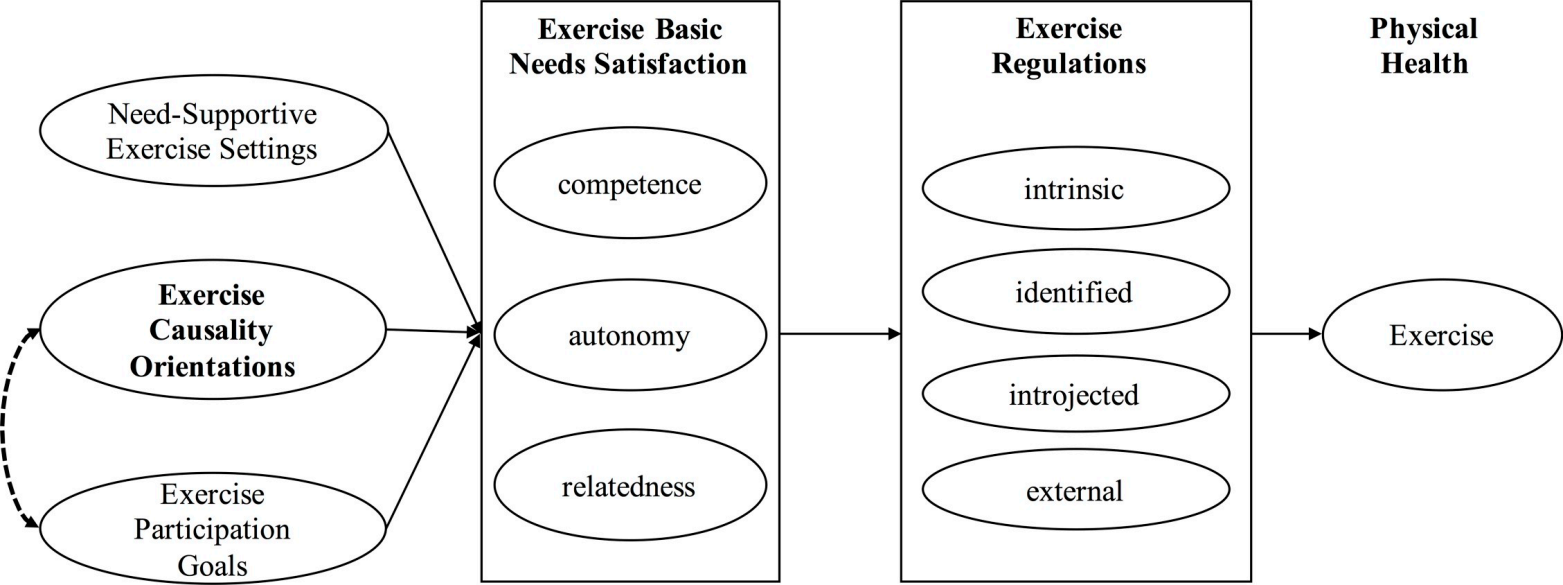
SDT process model for exercise behaviour (adapted from Teixeira et al., 2012). In this study, the connections between exercise causality orientations and exercise participation goals are to be explored (dashed line).

The SDT [5,6] is a theory focusing on individual differences in intrinsic and several forms of external motivation, human basic needs satisfaction, motives/life aspirations, and causality orientations. It is assumed that basic needs satisfaction leads to intrinsic forms of motivation and thus, to motivated behaviour. Basic needs satisfaction can be influenced by situational factors, motives/life aspirations, and causality orientations. The SDT has been applied to a wide range of contexts, including work, education, clinical psychology, and sport and exercise psychology [9–12]. Based on an application to the health context [13], the exercise basic needs satisfaction, exercise regulation modes, exercise participation goals, exercise causality orientations, and a situational component were integrated into a SDT process model for exercise behaviour [8] (Fig 1). Aligned with processes defined in the SDT model, these constructs have been successfully implemented to predict health behaviour adoption and maintenance from a motivational perspective [14–17], and have been examined across various cultures and countries [18,19]. Assessment tools measuring exercise specific regulation modes, basic needs satisfaction, and participation goals are available in English and German, and thus can be applied in the German speaking context (Table 1 for an overview). However, to date, an assessment tool measuring exercise causality orientations has only been provided in English [20], but not in German language to date.

**Table 1:** Summary of exercise specific measures related to the SDT health model.

Aspect in the SDT Health Model	Examined Relationships within the SDT Health Model	Frequently used measures	
		English language	German language
**Exercise Regulation Modes**	Relationships have been examined to all other aspects within the SDT Heath Model (each > 1 studies)	BREQ-3 [21]	SSK-Scale [22]
**Exercise Basic Needs Satisfaction**	Relationships have been examined to all other aspects within the SDT Health Model (each > 1 studies)	PNSES [23], BPNES [18]	PNSEG [24]
**Exercise Participation Goals**	Relationships have been examined to all other aspects within the SDT Health Model (each > 1 studies)	EMI-2 [25]	BMZI [26]
**Exercise Causality Orientations**	Relationships have only examined with Exercise Related Needs Satisfaction (one study)	ECOS [20]	*provided in this study*

#### Causality orientations

Causality orientations are a trait-like component of the SDT and can influence the interpretation of an event as informational, controlling, or amotivating. Hence, the level of causality orientations within a person can basically influence their motivational sequence [27,28]. Specifically, causality orientations reflect individual differences in assumptions about the causality of initiation and maintenance of behaviour [29]. Causality orientations can be divided into three aspects: autonomy orientation, control orientation, and impersonal orientation.

Autonomy orientation describes the assumption that behaviour is initiated and regulated by oneself. In this context, a situation is interpreted as informative, and thus can serve as a foundation of free choice and self-regulated behaviour. Consequently, autonomy orientation is likely to lead to perceived autonomy and competence need satisfaction, and intrinsic motivation, and therefore to high levels of self-determined behaviour and decisions.

Persons scoring high on control orientation expect their behaviour to be regulated by internal or external controls, such as rewards, gains or appreciation. Control orientation is hereby defined by interpreting situations as controlled, which is likely to promote extrinsic motivation (people believe they are told to) and introjected motivation (people believe they should).

Impersonal orientation describes the assumption that behaviour regulation lies beyond one’s own control, i.e., that behaviour and outcomes are independent from each other. Therefore, impersonal orientation is assumed to promote an interpretation of situations as amotivating. In this context, it has been found that impersonal orientation can lead to forms of learned helplessness [30].

In sum, causality orientations represent basic assumptions about the initiation and maintenance of behaviour. Moreover, causality orientations can be of high importance in explaining motivated behaviour as they fundamentally guide the motivational sequence of a person. All three causality orientations are each assumed to be prevalent to a certain degree in a person. Thus, causality orientations represent individual patterns instead of separate traits within an individual [29]. An exercise specific assessment tool measuring causality orientations has been provided in English language [28], but not in German language to date (Table 1).

#### Regulation modes

Regulation modes are a central aspect of the SDT [5,6] and vary on a continuum. In a general context, regulation modes reflect the inherence of motivation that is associated with behaviour: on the one side of the continuum, intrinsic motivation is characterized by inherent joy associated with the behaviour. More external regulations reflect engagement on the basis of congruence with personal goals (i.e., identified regulation) or the value of an outcome of behaviour (i.e., introjected regulation). On the other side of the continuum, external motivation is characterized by compliance to external controls, such as rewards or pressure. Exercise specific assessment tools measuring regulation modes have been provided in English and in German language [18,21,22].

#### Basic needs satisfaction

Additionally, three human basic needs are incorporated in the SDT: autonomy, competence, and relatedness. Satisfaction of these basic needs in a specific situation is the foundation for initiation of intrinsic motivation and thus, for motivated behaviour. Basic needs satisfaction is assumed to be influenced by situational factors (e.g., autonomy supporting environments), causality orientations, and specific goals/motives. Exercise specific assessment tools measuring basic needs satisfaction have been provided in English and in German language [23,24].

#### Participation goals

Exercise participation goals (sometimes also referred to as participation motives) describe an exercise specific motivation to initiate or to adhere to exercise behaviour. Exercise participation goals have been found to vary on their degree of intrinsic and external motivation [26]. Whereas activation/pleasure, aesthetics, contact, and competition/achievement have been associated with high intrinsic and low external regulations, distraction/catharsis and fitness/health have been associated with both intrinsic and extrinsic regulations. In contrast, appearance/physique goals have been associated with higher external and lower intrinsic regulations. Assessment tools measuring exercise participation goals have been provided in both English and German language [25,31].

#### Interrelations within the SDT process model

Within the SDT process model for exercise behaviour, the influence of need-supportive exercise settings and exercise participation goals on exercise basic needs satisfaction have been studied, meta-analysed, and reviewed comprehensively [7,8]. However, exercise causality orientations have been investigated in only few studies, providing little information about relations to other constructs in this model. The relationships between exercise causality orientations and exercise regulations have been examined in one study [16]. In this study, autonomy orientation was positively associated with intrinsic, identified and introjected regulations, and negatively associated with external regulation. Control orientation was unrelated to intrinsic and identified regulations, and positively associated with introjected and external regulations. Impersonal orientation was negatively related to intrinsic and introjected regulations, and positively associated with external regulation.

The relationships between exercise causality orientations and exercise basic needs satisfaction have not been examined yet. Moreover, evidence regarding interrelations between causality orientations and exercise participation goals in the health context has yet to be provided. Thus, further research is needed to investigate the interrelations between exercise causality orientations and other SDT related constructs to understand all the interrelations and processes within the SDT process model for exercise behaviour. Hence, by providing more elaborate insight into these motivational sequences, more accurate predictions of motivated behaviour, and specifically motivated health behaviour can be possible.

### Establishment of the original ECOS

In order to assess general causality orientations, the general causality orientations scale (GCOS) was developed originally [29]. The questionnaire comprises 36 items measuring the three scales autonomy, control, and impersonal causality orientation. In the questionnaire, situations from interpersonal, work, and informal context are presented in twelve vignettes. Each of the three interpretation options included in a vignette reflects one of the three scales. The GCOS questionnaire was initially validated on the basis of item-interrelations, indicating good indices and adequate stability. The vignettes were not tested with regards to the validity of their suggested factorial structure. However, the instrument was compared with a range of questionnaires measuring other SDT related constructs, personality questionnaires, and behaviour relations, such as exam grades. Causality orientations were assumed to be similar, but distinct from the concept of internal/external locus of control [32]: whereas locus of control describes contingency between behaviour and outcomes, causality orientations describe assumptions about initiation and maintenance of behaviour. Within the locus of control theory [32], internal control is characterized as an expectation that certain behaviour would lead to a certain reinforcement or outcome. Looking at different aspects of the assumptions about initiation, maintenance (i.e., causality orientations), and contingencies of behaviour (i.e., locus of control), autonomy and control orientation each cannot be clearly associated with one equivalent form of internal/external locus of control.

For a more specific measurement, the development of domain specific scales has been recommended [29]. Domain specific scales can be advantageous as they represent narrower classes of situations, and thus can be more suitable in predicting specific behaviour. An exercise specific scale has been provided with the ECOS [20], which also has a vignette format. The original version of the ECOS questionnaire includes nine vignettes in its first version and has been reduced to a seven-vignette version during an item selection (details see below). Every vignette contains three items, each representing one of the causality orientations. In the examination of its validity, a multi-trait multi-method approach was used, and confirmatory factor analyses (CFAs) were conducted. From a theoretical and statistical perspective, a correlated traits/correlated uniqueness (CTCU) model was preferred, inter-correlating the traits and all items within one vignette [33]. Thus, the residual terms of each vignette are likely to capture specific variance that is due to the situation described in a vignette [34]. Because the CFA with nine vignettes did not yield an acceptable model fit, the questionnaire was reduced to a seven-vignette version on the basis of a stepwise inspection of factor loadings and examination of the new model fit. The results of the final seven-vignette version indicated a sufficient model fit. The coefficients for internal consistency ranged from .65 ≤ Cronbach’s *α* ≤ .70, which can be assumed to be large considering the vignette technique [35]. Examining the concurrent validity of the ECOS, autonomy orientation was correlated with intrinsic regulation modes, whereas control and impersonal orientation were correlated with external regulation modes [20]. In a second validation study, autonomy orientation was related to higher levels of intentions and actions to change behaviour for exercise context [16].

### The present work

With a special focus on individual differences in the assumptions about the initiation and maintenance of behaviour, exercise causality orientations are part of the SDT process model for exercise behaviour [8,20]. The assessment of exercise causality orientations can be a key element to improve predictions of motivated health and exercise behaviour. Nevertheless, a scale to measure exercise causality orientations has not been established in German yet. Also, exercise causality orientations have scarcely been associated with other SDT related aspects, such as exercise participation goals. Thus, it was the aim of this work to translate the ECOS [20] to German, to test this questionnaire across several samples, and thus to complement the SDT related assessment tools that are available in German (Table 1). Hence, it was the aim to contribute to internationally valuable theory development with regards to the broadly applied SDT process model for exercise behaviour. In doing so, construct and concurrent validity were comprehensively examined via three studies. In addition, the interrelations of the exercise causality orientations with SDT related aspects were tested to extend the knowledge within the SDT process model for exercise behaviour.

## Study 1: Preparation of the G-ECOS

It was the aim of Study 1 to translate the ECOS to German language and to test the questionnaire in a first sample. Using the nine original vignettes as a starting point, it was another aim to conduct a selection of vignettes that was analogue to the procedure conducted in the original ECOS study. The study has been approved by the ethics committee of the University of Münster.

### Material and methods

To guarantee high quality and validity of questionnaires that are adapted to other cultures and languages, a guideline for the translation and validation of questionnaires has been proposed [36]. In this context, several points are targeted: first, the original questionnaire should provide sufficient reliability and validity, and potential copyright questions should be checked. Second, it has been recommended that details of the translation and back-translation process are accessible. Third, differences in measurement inequivalence are likely to occur, and can be due to cultural differences. These inequivalences do not endanger the quality of a translated questionnaire per se, given that explanations for the differences can be identified. Fourth, translated questionnaires should be cross-validated, preferably in independent and broader samples. Fifth, the translated version should be further validated via the analysis of associations with external criteria. Thus, to provide a valuable and high-quality translation of the ECOS into German, these guidelines were followed throughout the three studies included in this work.

#### Study design

In Study 1, the original vignette-based ECOS questionnaire was translated to German and tested in a first sample. Thus, in a first step, a forward-backward translation of the ECOS questionnaire was conducted in cooperation with a native speaker. In particular, the authors—who were non-native speakers—translated the questionnaire, which was then back-translated by a bilingual native speaker. Afterwards, the original English version and the back-translated version of the questionnaires were compared and discussed by the native speaker and the author. The native speaker and the author agreed that no substantial differences in the meaning of the items were observable. Minor differences in the English wording of the translated questionnaire were adjusted afterwards. All details of the translation and back-translation process including the adjustments are provided in the supplements (S1 and S2 Tables). The original ECOS questionnaire entailed nine vignettes, each describing a situation in the exercise setting. Thus, all nine vignettes were translated and were used as a starting point of the item selection, analogously to the ECOS study. Every vignette comprises three items, each representing one of the three scales autonomy, control, and impersonal orientation. The participants were asked to rate each of the answers on a seven-point Likert scale (1 = *very unlikely* to 7 = *very likely*).

#### Participants and data collection

A total of *N* = 306 students (72.60% female) was recruited via personal contact and flyers from sport science or psychology classes. The participants filled in the translated version of the questionnaire, i.e., the German Causality Orientations Scale (G-ECOS) via paper-pencil questionnaires. An estimation of the required sample size was conducted via an online tool that provides sample size calculation for structural equation models [37]. The anticipated effect size was based on the original ECOS model [28]. Thus, defining a desired statistical power level of .80, a probability level of *α* = .05, a number of nine latent variables, and 27 observed variables, it was estimated that a minimum sample size for the model structure was *n* = 200, and that a minimum sample size to detect an effect of .30 was *n* = 184. Participants who were 18 years or older were included in the study. Besides, no inclusion or exclusion criteria were defined. All participants included in the study gave their informed consent on the questionnaire in written form via an item included in the questionnaire. On average, the participants were aged 26.00 years (*SD* = 5.66). Data sets were included in the analysis if at least 21 of the 27 items (according to two out of three scales) had been answered. Thus, the data of one participant was excluded.

#### Data analysis

In a first step, the descriptive results of Study 1 were compared to the descriptive data obtained from the original ECOS. The raw data set was provided by the authors of the original ECOS questionnaire [20]. Part-whole-corrected item-scale correlations were calculated. In a second step, a CFA was conducted via a multi-trait, multi-method approach [33] that was comparable to the original validation of the ECOS questionnaire. Data was inspected via Mardia tests for normality, skewness, and kurtosis [38]. Normalized Mardia coefficients were *χ*^2^ = 6086.90 for skewness (*p* < .001) and *z* = 20.47 for kurtosis (*p* < .001). Thus, the assumptions of multivariate normality were not met, and the robust scaled estimator MLM [39] was applied in the CFA. Analogous to the original validation study, and according to general assumptions [34], it was assumed that a CTCU model would be appropriate to represent the vignette format. Thus, the initial nine vignettes were used to compare a CTCU model vs. a correlated traits model (CT). A correlated traits/correlated methods model (CTCM) and a correlated traits/uncorrelated methods models (CTUM) were not considered as they could not be generated. In a first step, the 7-vignette solution of the original ECOS questionnaire was tested. If an inacceptable model fit was indicated, the original 9-vignette solution of the ECOS was used as a starting point of the stepwise reduction of vignettes, as it had been conducted in the original ECOS study. During the modelling process, non-fitting vignettes were removed stepwise from the model. The removal criterion was defined as the smallest associations with the related factor or the highest associations with unrelated factors. Stopping removal of vignettes was guided by two criteria: (1) an acceptable model fit was indicated: CFI > .90, RMSEA < .08 [40]; (2) Δ*χ*^2^ of a model and the previous model did not indicate a significant difference in *χ*^2^ indices.

With regards to reliability, the models of each scale were first tested for meeting the assumptions of equal factor loadings that are required for the use of Cronbach’s *α* [41]. The congeneric model fitted the data better than the essentially tau-equivalent model. Therefore, the estimation via Cronbach’s *α* might have led to an overestimation of coefficients [35,42], and McDonald’s *ω*_*H*_ [43,44] was used to calculate the reliability. Due to the nature of vignette studies that apply highly situation specific content, acceptable reliability is already indicated by *ω*_*H*_ > .50 [35]. The statistical data analyses were conducted via the program *R* [45], using the packages *lavaan* [46] and *psych* [47], and via the program *JASP* [48]. To provide transparency of the analyses of this work and to make this research verifiable and reproducible, the code used in this study, the data set, the codebook and the translated items are provided online at the open science framework OSF: https://osf.io/ew6g7/?view_only=daa1ea1cb3f34ef593099659bd9496d8

### Results

**Fig 2:**
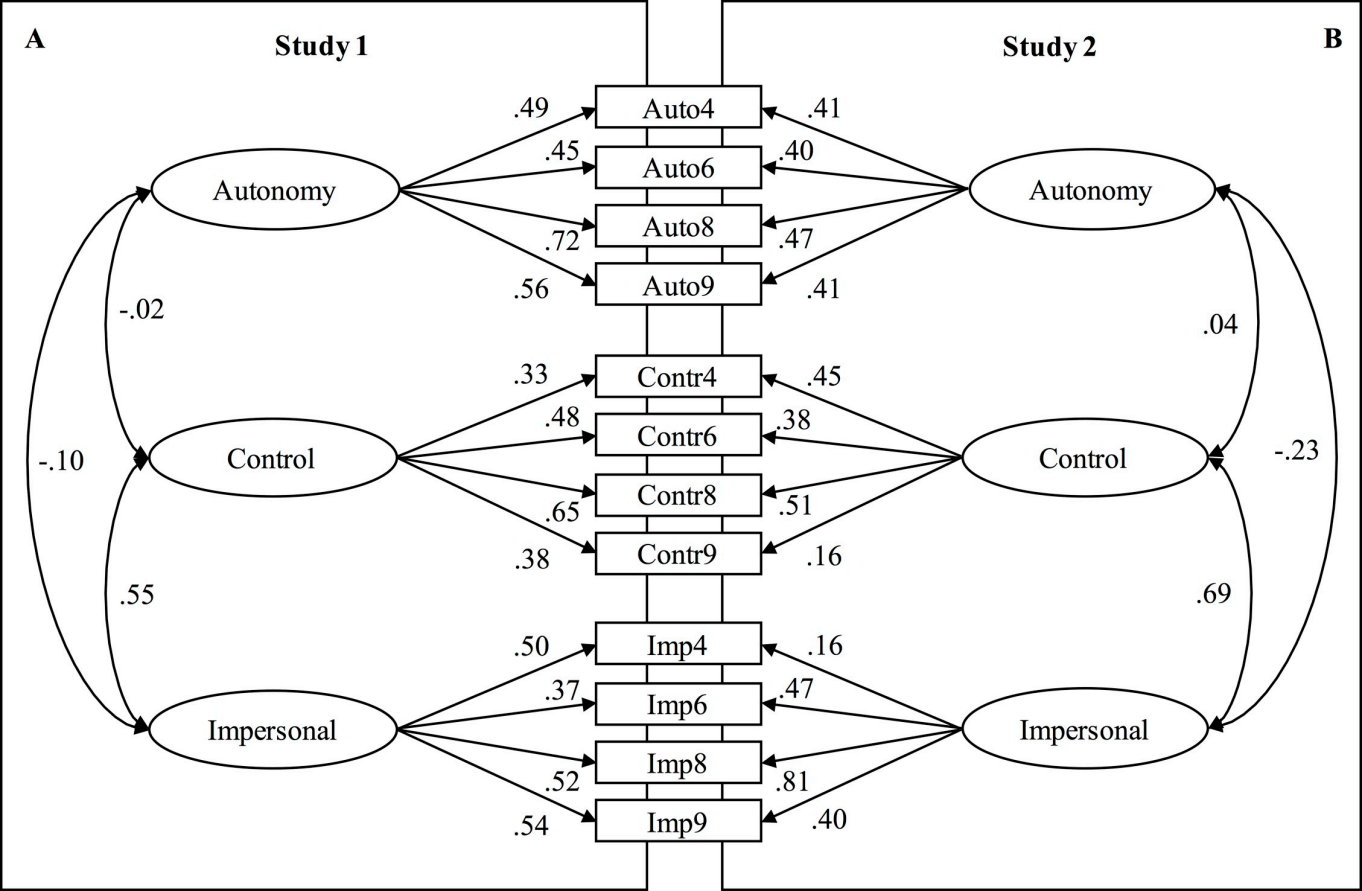
Factor loadings in Study 1 and Study 2 in the final vignettes 4, 6, 8, 9. Auto4 = Item measuring Autonomy scale in vignette 4.

In Study 1, the methodology of the original questionnaire was replicated, examining the factorial structure of the translated G-ECOS questionnaire. Means and standard deviations for the reduced four-vignette questionnaire were 5.36 for autonomy (*SD* = 0.28), 4.41 for control (*SD* = 0.40) and 3.83 for impersonal orientation (*SD* = 0.27). Part-whole-corrected item-scale-correlations were .40 ≤ *r*_*it(i)*_ ≤ .50 for autonomy, .22 ≤ *r*_*it(i)*_ ≤ .44 for control, and .29 ≤ *r*_*it(i)*_ ≤ .40 for impersonal orientation (Table 2). Standardized factor loadings were .45 ≤ *λ* ≤ .72 for autonomy, .33 ≤ *λ* ≤ .65 for control, and .37 ≤ *λ* ≤ .54 for impersonal orientation (Fig 2A).

**Table 2:** Item characteristics of the final vignettes 4, 6, 8, 9 in Study 1 (left side) and of the scales in the original and present validation studies.

Scale in Study 1	Item/Vignette	*M*	*SD*	Skewness	Kurtosis	*r*_*it(i)*_	Scale and Study	*M*	*SD*	*σ*
Autonomy	Auto4	5.10	1.34	–0.87	0.62	.41	Autonomy			
	Auto6	5.30	1.63	–0.95	0.08	.40	**ECOS** Original	5.19	1.07	.74
	Auto8	5.28	1.31	–1.02	0.99	.50	**GECOS**, Study 1	5.36	0.28	.77
	Auto9	5.75	1.32	–1.46	2.30	.41	**GECOS**, Study 2	5.56	0.22	.79
Control	Contr4	4.21	1.55	–0.30	–0.78	.22	Control			
	Contr6	3.96	1.63	0.00	–0.78	.34	**ECOS** Original	3.99	1.02	.57
	Contr8	4.63	1.43	–0.46	–0.26	.44	**GECOS**, Study 1	4.41	0.40	.63
	Contr9	4.83	1.58	–0.69	–0.20	.29	**GECOS**, Study 2	4.72	0.46	.67
Impersonal	Imp4	3.52	1.63	0.25	–0.80	.31	Impersonal			
	Imp6	3.28	1.26	0.32	–0.34	.29	**ECOS** Original	3.08	1.10	.44
	Imp8	3.06	1.56	0.51	–0.60	.40	**GECOS**, Study 1	3.83	0.27	.55
	Imp9	3.67	1.80	–0.02	–1.16	.33	**GECOS**, Study 2	3.50	0.29	.50

*r*_*it(i)*_ part-whole-corrected item-scale-correlations. Auto4 = Item measuring Autonomy scale in vignette 4. ECOS Original *n* = 561; GECOS Study 1 *N* = 306, Study 2 *N* = 268. *σ* = Item difficulty *M*/7, based on the seven-point scale.

When comparing a CT vs. a CTCU model, the CTCU model indicated a better model fit (Δ*χ*^2^ [27] = 186.55, *p* < .001; see S3 Table for all model comparisons). Thus, analogously to the original ECOS study, a CTCU model that is defined by correlated error terms was used for the analysis. In the replication of the original seven-vignette ECOS questionnaire, an insufficient model fit was indicated (*N* = 306; *χ*^2^ = 293.90, *df* = 165, *p* < .001; CFI = .83; TLI = .78: SRMR = .08; RMSEA = .06, 90%-CI [.05; .07]). Also, the results of the CFA indicated an insufficient model fit of the nine-vignette questionnaire. During the stepwise reduction of items, an acceptable model fit could be first identified in a model comprising the four vignettes 4, 6, 8, and 9 (*N* = 306; *χ*^2^ = 54.05, *df* = 39, *p* = .055; CFI = .96; TLI = .93; SRMR = .05; RMSEA = .04, 90%-CI [.00; .06]; Table 3). Further reduction of a vignette did not lead to a substantial improvement of the model fit, indicated by an insignificant change in Δ*χ*^2^. The model fit indices for each step of exclusion of the vignettes are provided in Table 3. The four-vignette solution was also applied to the original ECOS data that had been provided by the original authors. Here, the four-vignette version displayed an insufficient model fit (*N* = 594; *χ*^2^ = 715.21, *df* = 51, *p* < .001; CFI = .52; TLI = .93; SRMR = .13; RMSEA = .17, 90%-CI [.16; .18]).

**Table 3:** Model fit indices for each step of exclusion of vignettes.

Step	Model	Exclusion of vignettes	Scaled *χ*^2^	*df*	*p*	CFI	TLI	SRMR	RMSEA	90%-CI for RMSEA	Δ*χ*^2^ of comparison with previous model	Δ*χ*^2^*df*	Δ*χ*^2^*p*
	9 vignettes		524.05	294	< .001	.81	.77	.09	.06	.05; .07			
1	8 vignettes	*vignette 2*	378.11	225	< .001	.85	.81	.08	.05	.04; .06	144.83	69	< .001
2	7 vignettes	*vignette 7*	271.86	165	< .001	.86	.82	.07	.05	.04; .06	106.42	60	< .001
3	6 vignettes	*vignette 1*	189.74	114	< .001	.89	.85	.07	.05	.04; .07	81.83	51	.004
4	5 vignettes	*vignette 5*	131.79	72	< .001	.89	.83	.06	.06	.04; .07	58.76	42	.044
5	4 vignettes	*vignette 3*	54.05	39	.055	.96	.93	.05	.04	.00; .06	77.83	33	< .001
6	3 vignettes	*vignette 6*	20.36	15	.16	.97	.93	.04	.04	.00; .07	33.67	24	.091

Inter-correlations of the scales were (1) *r* = -.02 for autonomy and control orientation, (2) *r* = -.10 for autonomy and impersonal orientation, and (3) *r* = .55 for control and impersonal orientations. Regarding the estimation of reliability, the congeneric model fitted the data better (Δ*χ*^2^ [11] = 22.84, *p* = .019). Reliability coefficients were *ω*_*H*_ = .65 (*α* = .66) for autonomy, *ω*_*H*_ = .56 (*α* = .53) for control, and *ω*_*H*_ = .56 for impersonal orientation (*α* = .54).

### Discussion

Exercise causality orientations reflect basic assumptions about the initiation and maintenance of health-related behavior that are assumed to guide the motivational sequence. Thus, the assessment and consideration of exercise causality orientations can serve to better understand motivated exercise behavior [27,28]. In this context, the translation of validated questionnaires are often required to contribute to valuable and comparable research across cultures and languages [36]. Thus, it was the aim of Study 1 was to develop and validate a German version of the ECOS questionnaire measuring exercise causality orientations. Particularly, it has been suggested that the original questionnaire provides sufficient reliability and validity, and potential copyright questions should be checked [36]. The ECOS is a well-established and validated questionnaire measuring exercise specific causality orientations that are incorporated in the widely and successfully used SDT [6,28,49,50]. Furthermore, according to the guidelines [36], details of the process of translation and back-translation were recommended to be accessible. In this study, all details regarding the translation process were provided, and complimentary comprehensive supplementary material is available. Furthermore, the raw data set of the ECOS study was provided by the original authors and was used for elaborate comparisons with this study. Thus, with regards to the guidelines, all criteria for valid and high-quality translations were met in this study. In the examination of the model fit and the reliability in this study, good coefficients were found, indicating evidence for the validity of the German G-ECOS. The questionnaire was validated analogously to the methodology used in the validation of the original ECOS questionnaire [20]. Based on an original pool of nine vignettes—that had been the starting point in both this and the ECOS study—a stepwise removal of vignettes led to a good model fit of a final four-vignette version of the G-ECOS. In the G-ECOS, the vignettes 4, 6, 8, and 9 were included, whereas the original ECOS comprises the vignettes 1, 3, 4, 5, 7, 8, and 9. Based on the guidelines for the translation of questionnaires, it has been indicated that differences in measurement inequivalence (i.e. identification of different item selection during the statistical validation) are likely to occur during the translation of questionnaires [36]. However, this do not endanger the quality of a translated questionnaire per se. In the case of inequivalent measures, it is rather required to recognize explanations for the differences in the questionnaires [36]. Possible explanations could be identified as follows: (1) in contrast to the equivalent items, all *non-equivalent* vignettes 1, 3, 5, 6, and 7 describe situation in fitness instructor or fitness centre contexts. Here, cultural differences in the setting of such situations (e.g., how fitness centres and courses are structured in university context across Great Britain and Germany) might have led to inequivalence of item selection; (2) the populations assessed might have experienced different settings of fitness centres and instructors (i.e. the German sample regularly attends sport classes at the university whereas the British non-student sample attends a gym during leisure time); (3) the German G-ECOS samples comprised sport science students aged 26 years on average, whereas the ECOS sample comprised university staff and employees of private companies aged 35.8 years on average. Thus, it was an aim of the subsequent Study 2 to cross-validate the G-ECOS in an independent and broader sample.

All GCOS, ECOS and the current G-ECOS are based on a vignette format, each entailing three items corresponding to the three causality orientations. The usage of vignettes to measure personality traits can be advantageous as they comprise concrete and natural situations, and thus are likely to provide high content validity. Nevertheless, vignette measurement is situation specific, potentially leading to high specific variance in item total variance. The application of a multi-trait-multi-method approach [33] is one approach to consider this specific variance in the CFA. However, when applying vignettes, an item selection can lead to a radical reduction of the item number: Once one item of a vignette is regarded inappropriate, the entire vignette entailing three items has to be deleted. Coefficients measuring item consistency (i.e., Cronbach’s *α*; *ω*_*H*_) might stand in contrast to the approach of this study using vignettes and focusing on high content validity [51]. Overall, it should also be noted that short inventories often do not provide sufficient accuracy for individual-based diagnostic purposes. Specifically, scores on very brief scales and vignettes may be valid, but can at the same time lack a certain amount of measurement precision (reliability) that is needed for individual-level assessment [52]. Furthermore, individual-level implications may suffer more from scale shortening compared to implications on group-level decisions [53]. Therefore, we caution practitioners against the use of this scale for purposes of *individual* assessment until evidence for its reliability and validity in according settings and beyond group-level research has been obtained. In sum, a German translation of the established original ECOS questionnaire showing a valid four-vignette structure characterized by good fit indices was provided in Study 1. To ensure high-quality and valuable translation and validation of questionnaires, it has also been recommended to cross-validate the translated versions [54]. Thus, Study 2 was designed to test the G-ECOS in an independent and broader sample.

## Study 2: Cross-validation of the G-ECOS

As the German four-vignette questionnaire G-ECOS has been established in Study 1, it was the aim of Study 2 to cross-validate the G-ECOS questionnaire in a second and independent sample. In doing so, the four vignettes identified in Study 1 were used. It was another purpose to distribute the questionnaires to a broader population than in Study 1 in order to enhance the validity. Additionally, a broader multi-format methodology of both online and paper pencil questionnaire was used in Study 2 compared to the paper-pencil format used in Study 1. The study has been approved by the ethics committee of the University of Münster.

### Material and methods

In Study 2, an independent sample comprising *N* = 320 participants (70.94% female) was recruited via personal contact, flyers, and social networks. The data collection was conducted via a paper-pencil version that was distributed at university classes via personal contact and flyers. The questionnaire was filled in by students mostly enrolled in sport science and psychology classes (*n* = 106). One similarly designed online version of the questionnaire (i.e., with regards to the item presentation and font) was created and provided via the online survey program *unipark*. The online link was distributed via personal contact, flyers and social networks, and it was completed by *n* = 121 participants. All participants filled in the four-vignette version of the G-ECOS questionnaire that had been established in Study 1. An estimation of the required sample size was conducted via an online tool that provides sample size calculation for structural equation models [37]. The anticipated effect size was guided by the results found in Study 1. Defining a desired statistical power level of .80, a probability level of *α* = .05, a number of four latent variables and 12 observed variables, it was estimated that a minimum sample size for the model structure was *n* = 200, and that a minimum sample size to detect an effect of .30 was *n* = 137. Again, participants who were 18 years or older were included in the study and gave their informed consent in written form via an item included in both the paper-pencil and the online questionnaires. Persons participating in this data collection were aged 29.00 on average (*SD* = 3.54). Eighty-three per cent of the participants in Study 2 were students, including 23% psychology students and 35% sport science students. Data sets were included in the analysis if at least eight of the twelve items had been answered (thus, again according to two out of three scales). The final data set of the sample in Study 2 entailed the answers of *N* = 268 participants.

#### Data analysis

The factorial structure of the G-ECOS was analysed via a CFA. The model specifications followed the same steps as in Study 1. Normalized Mardia coefficients were *χ*^2^ = 737.16 for skewness (*p* < .001) and *z* = 8.37 for kurtosis (*p* < .001), indicating multivariate non-normality. Thus again, the robust scaled estimator MLM [39] was applied. The statistical data analyses were conducted via the program *R* [45] using the packages *lavaan* [46] and *psych* [47], and via the program *JASP* [48]. The data and code used in this study are provided online at the open science framework OSF: https://osf.io/ew6g7/?view_only=daa1ea1cb3f34ef593099659bd9496d8

### Results

In Study 2, the G-ECOS questionnaire entailing the four vignettes identified in Study 1 was cross-validated. Means were 5.56 for autonomy (*SD* = 0.22), 4.72 for control (*SD* = 0.46), and 3.50 for impersonal orientation (*SD* = 0.29), as summarized in Table 2. The results of the CFA again indicated a good model fit (*N* = 268 *χ*^2^ = 50.15, *df* = 39, *p* = .109; CFI = .95; TLI = .92 SRMR = .05; RMSEA = .04, 90%-CI [.00; .06]). Standardized factor loadings and interrelations are presented in Fig 2B. Regarding the estimation of reliability, the congeneric model fitted the data better (Δ*χ*^2^ [11] = 47.41, *p* < .001). Thus, *ω*_*H*_ should be interpreted. Reliability coefficients were *ω*_*H*_ = .50 (*α* = .49) for autonomy, *ω*_*H*_ = .41 (*α* = .37) for control, and *ω*_*H*_ = .52 (*α* = .45) for impersonal orientation. Compared to the original ECOS, the interrelations between the constructs were (1) autonomy and control: *r* = -.02 and *r* = .04 in G-ECOS studies, *r* = .01 in ECOS; (2) autonomy and impersonal: *r* = -.10 and *r* = -.23 in G-ECOS studies, *r* = -.53 in ECOS; and (3) control and impersonal: *r* = .55 and *r* = .69 in G-ECOS studies, *r* = .55 in ECOS). Overall, the item means were higher in the G-ECOS studies compared to the ECOS study.

### Discussion

Setting a guideline for translations of questionnaires in other languages, it has been recommended to cross-validate a translated questionnaire [36]. Thus, it was the aim of Study 2 to cross-validate the G-ECOS in an independent and broader sample. In the examination of the factor structure, the good factorial validity identified in Study 1 could be replicated, indicating that the G-ECOS is a robust and valid assessment tool. Specifically, most factor loadings demonstrated similar factor loadings within the two studies, indicating a robust factor structure. However, few items showed lower loadings, indicating that further validation studies are needed. The provided G-ECOS shows theory-conform interrelations between autonomy, control and impersonal causality orientations. Correspondingly, coefficients of item difficulty were also higher in the G-ECOS studies than in the ECOS study. These results indicate that the German sample tended to agree to all statements to a higher extent. Also, lower reliability coefficients were found in Study 2 compared to Study 1. These results might be an artefact of the more heterogeneous sample that was assessed in Study 2. Furthermore, a combination of a paper-pencil version and an online version was used in Study 2 compared to a paper-pencil version that was applied in Study 1. In this context, it had been an aim to standardize the item layout and formatting of the paper-pencil version and the online version. However, minor and inevitable differences in the layout and context (i.e., in the classroom vs. at home) could have affected the participants’ responses.

In sum, a translation, validation, and cross-validation of the established original ECOS has been provided in Study 1 and Study 2 that has been conducted on the basis of two independent data sets. Overall, the G-ECOS can be regarded as a valid and useful tool complementing the SDT related and validated instruments that are available in German language. Building up on these results, it was the aim of Study 3 to connect exercise causality orientations with other SDT related aspects. Thus, it was the purpose to better understand how differences in causality orientations are related to motivational aspects explaining the sequence of motivated exercise behaviour.

## Study 3: Relations within the SDT process model for exercise behaviour

The SDT proposes a range of sub-theories describing and explaining a sequence of human motivated behaviour, including specific health related and exercise behaviour. The exercise causality orientations represent individual differences that guide the interpretation of situations as informational, controlling, or amotivating, and thus can basically guide the motivational consequences [28]. However, the interrelations between exercise causality orientations and other SDT related aspects, such as regulation modes, basic needs satisfaction, and exercise participation goals have scarcely been targeted in previous studies. Thus, to better understand how differences in causality orientations are related to motivational aspects, it was the aim to test these relations in this study. Following the guidelines for high quality and valuable validation of translated questionnaires [54], it was also the aim to elaborate on the associations between the G-ECOS questionnaire and external criteria, such as related constructs incorporated in the SDT.

Based on theoretical assumptions and the results found in previous studies, it was hypothesised:

*Hypothesis 1*: High levels of autonomy causality orientations are positively associated with intrinsic motivation regulation, whereas high levels of control causality orientations are positively associated with extrinsic forms of motivation regulation.

*Hypothesis 2*: High levels of autonomy causality orientations are positively associated with autonomy and competence need satisfaction, whereas high levels of control causality orientations are negatively associated with exercise basic needs satisfaction.

*Hypothesis 3*: High levels of autonomy causality orientations are positively associated with intrinsic forms of exercise participation goals, whereas high levels of control causality orientations are positively associated with extrinsic exercise participation goals.

### Methods

A total of *N* = 548 adults (62.50% female) participated in Study 3. As the tested model was identical to the analysis in Study 2, the sample size estimation again yielded a minimum sample size for the model structure of *n* = 200. The participants in the third data collection were aged 30.17 years on average (*SD* = 11.91). Participants who were at least 18 years old were recruited via personal contact, mailing lists, and social networks, focusing on students and sport club participants. All participants included in the study gave their informed consent via an item included in the online questionnaire. The data collection was conducted via an online questionnaire that was identical to the questionnaire used in Study 2. The questionnaire was provided via the online survey program *unipark*, not allowing to skip questions. The study has been approved by the ethics committee of the University of Münster.

#### Exercise causality orientations

Autonomy, control, and impersonal orientation (each four items) were assessed using the G-ECOS that had been established throughout Study 1 and Study 2. Each item was rated on a seven-point Likert scale. In this study, both Cronbach’s *α* and *ω*_*H*_ can be interpreted as the essentially *τ* -equivalent model did not fit the data better (Δ*χ*^2^[11] = 18.92, *p* = .062). Reliability was *ω*_*H*_ = .57 (*α* = .57) for autonomy, *ω*_*H*_ = .53 (*α* = .52) for control, and *ω*_*H*_ = .47 (*α* = .45) for impersonal orientation.

#### Exercise regulations

Exercise regulations are an exercise specific concept embedded in the SDT and the self-concordance model [55]. Exercise regulations reflect the concordance of a goal with personal values and interests. In this study, the German assessment tool for measuring the self-concordance of sport- and exercise-related goals SSK-Scale [22] was applied. The SKK-scale consists of twelve items, measuring the scales intrinsic, identified, introjected, and extrinsic regulations (each three items) on a six-point Likert scale. The essentially *τ* -equivalent model fitted the data better (Δ*χ*^2^ [11] = 263.38, *p* < .001), and thus *ω*_*H*_ should be interpreted. In this study, reliability was .75 ≤ *ω*_*H*_ ≤ .83 (.72 ≤ *α* ≤ .81). Validity tests have indicated the instrument to be robust [22].

#### Exercise basic needs satisfaction

To assess exercise basic needs satisfaction, the German psychological needs satisfaction in exercise scale [56] was used. The assessment tool entails eleven items. The three scales autonomy (three items), competence (four items) and relatedness (four items) were assessed on a seven-point Likert scale. Reliability and validity have been shown to be satisfactory [56]. In this study, the essentially *τ* -equivalent model fitted the data better (Δ*χ*^2^ [10] = 113.20, *p* < .001), and thus *ω*_*H*_ should be interpreted. Reliability was .75 ≤ *ω*_*H*_ ≤ .89 (75 ≤ *α* ≤ .88; Cronbach’s *α* is presented to facilitate comparisons with the original study).

#### Exercise participation goals

In order to measure exercise participation goals, the Bernese motive and goal inventory in leisure and health sports BMZI [26] was applied. The BMZI consists of 24 items that are rated on a five-point Likert scale. Scales are fitness/health (3 items), appearance/physique (3 items), distraction/catharsis (3 items), activation/pleasure (3 items), aesthetics (2 items), competition/achievement (4 items), and contact (5 items). In this study, the essentially *τ* -equivalent model fitted the data better (Δ*χ*^2^ [23] = 469.37, *p* < .001), and thus *ω*_*H*_ should be interpreted. Reliability was .73 ≤ *ω*_*H*_ ≤ .92 (.68 ≤ *α* ≤ .92; Cronbach’s *α* is presented to facilitate comparisons with the original study). Evidence towards construct and concurrent validity has been provided [26].

#### Data analysis

To examine the interrelations between exercise causality orientations and the SDT related exercise participation goals, exercise regulation modes, and exercise basic needs satisfaction, Bayesian correlations with stretched beta prior width 1 and 95% credible intervals were estimated. The prior was chosen to be 1, because no relevant theoretical background information indicated to changing the prior. Bayes factors above 30 can be interpreted as very strong effects, above 10 as strong effects and above 3 as moderate effects [57]. A Bayes factor above 1 provides evidence towards the alternative hypothesis whereas as Bayes factor below 1 represents evidence in favour of the null hypothesis. A Bayes of ten in favour of the alternative hypothesis means, that the alternative hypothesis predicts the data ten times better than the null hypothesis. Statistical data analysis was conducted via JASP [48]. The data and code used in this study are provided online at the open science framework OSF: https://osf.io/ew6g7/?view_only=daa1ea1cb3f34ef593099659bd9496d8

### Results

The relationships between all SDT related variables assessed in this study (i.e., exercise causality orientations, exercise need satisfaction, exercise regulation and exercise participation goals) are presented in S4 Table. In the examination of exercise regulation modes and exercise causality orientations, medium positive correlations were found between autonomy orientation and intrinsic and identified regulations. A small negative correlation was found between autonomy and external regulation. Regarding control orientation, a small positive correlation was found with identified motivation, and medium positive correlations were found with introjected and external regulations. Impersonal orientation was negatively correlated with intrinsic regulation to a small extent. Medium positive correlations were found between impersonal orientation and introjected and extrinsic regulations. Regarding basic needs satisfaction, medium positive correlations were found between autonomy orientation and autonomy and competence needs satisfaction, and a small positive correlation was found with relatedness need satisfaction. Regarding impersonal orientation, small negative correlations were found with autonomy and competence needs satisfaction.

With regards to the relationships between exercise participation goals and exercise causality orientations, medium positive correlations were found between autonomy orientation and fitness/health, activation/pleasure, and aesthetics. Small positive correlations were found between autonomy orientation and distraction/catharsis, and competition/achievement. For control orientation, small correlations were found with fitness/health, appearance/physique, distraction/catharsis, and competition. Regarding impersonal orientation, small positive correlations were found with appearance/physique, and distraction/catharsis. A negative small correlation was found between impersonal orientation and activation/pleasure.

### Discussion

It was the aim of Study 3 to test the relations between the exercise causality orientations and related motivational aspects incorporated in the SDT. Thus, it was the purpose to better understand how differences in exercise causality orientations are associated with the motivational sequence postulated in the SDT. Following the guidelines for high quality and valuable validation of translated questionnaires [54], it was also the aim to elaborate on the associations between the G-ECOS questionnaire and external criteria. Overall, this study provides further contribution to theory building of the SDT process model for exercise behaviour.

#### Exercise regulation modes

In this study, autonomy orientation was positively related to intrinsic forms of motivation and control, and impersonal orientation was positively related to external forms of motivation. Therefore, *Hypothesis 1* was confirmed. The results indicate that autonomous control orientation is the equivalent to autonomous motivation, which are both assumed to lay inherent in the person. In contrast, control and impersonal orientated behaviour are assumed to originate from external causes. Based on theoretical assumptions, causality orientations influence the interpretation of a situation as informational, controlling, or amotivating [27,28]. As a consequence, high levels of autonomy causality orientations can promote intrinsic motivation, whereas high levels of control orientations can promote extrinsic forms of motivation, and impersonal causality orientations can promote amotivation. In sum, the results found in this study are in line with the theoretical assumptions and the results reported in ECOS validation studies [16,20]. Beyond the background of the SDT process model for exercise behaviour, it can be assumed that the associations between exercise causality orientations and regulation modes are mediated via exercise basic needs satisfaction.

#### Exercise basic needs satisfaction

In this study, autonomy orientation was positively related to all three aspects of basic needs satisfaction, and especially to autonomy and competence need satisfaction. Thus, *Hypothesis 2* was confirmed. Autonomy orientation has been described as engagement based on own interests and assumption that behaviour is initiated by oneself. Those assumptions might make a person likely to seek for situations in which a person can experience autonomy, competence, and relatedness. The results found in this study are also in line with previous results found in studies conducted in the work context, finding that autonomy control orientation is associated with autonomy need satisfaction [58]. Furthermore, control orientation was not related to exercise basic needs satisfaction in this study. Control orientation has been described as an engagement that origins from the initiation by others and by focus on external controls. Thus, it might be unlikely to experience autonomous and competence need satisfaction in such situations. Hence, it is in line with theoretical assumptions that no associations between control orientation and exercise basic needs satisfaction were found. In the analysis of impersonal orientations, negative relations with exercise basic needs satisfaction were found, specifically with autonomy and competence needs satisfaction. Hence, persons who are not motivated by either autonomous or controlled forms might be unlikely to seek for exercise related situations in general. Thus, these persons might be less likely to experience exercise related needs satisfaction. Beyond the background of the SDT process model for exercise behaviour, it can be assumed that interindividual differences in assumptions about the initiation of behaviour can lead to different levels of basic needs satisfaction. However, it stays unclear how the interindividual differences can also influence the evaluation and perception of contextual factors or the specific goal setting. In this context, potential interaction effects between exercise causality orientations and exercise participation goals/contextual factors on exercise basic needs satisfaction might be prevalent and should be addressed in further studies.

#### Exercise participation goals

Regarding the relationships between exercise causality orientations and exercise participation goals, a strong connection between autonomy orientation and intrinsic goals was found. Beyond the theoretical background, both aspects imply the presence of high inherent and self-determined motivation. The results indicate that the initiation of action associated with intrinsic goals (e.g., to compete with others or to have fun) is likely to be originated within the person.

The findings also indicate the presence of a connection between control orientation and external goals. Analogously, the results indicate that behaviour that is related to external exercise goals is often initiated by others (e.g., via social pressure in training groups). Furthermore, in this study, impersonal orientation was positively associated with external participation goals, but also with contact goal. These findings are in line with theoretical assumptions postulating that external goals do not reflect behaviour that is initiated in a self-determined fashion. The same process might be assumed when persons are participating in groups and perceive that the initiation and maintenance of their exercise behaviour is controlled by others. Thus, in sum, *Hypothesis 3* could be confirmed.

Overall, Study 3 provides evidence towards the presence of relationships between exercise causality orientations and exercise basic needs satisfactions, and exercise participation goals. Regarding the interrelations found between exercise causality orientations and exercise participation goals, the results from this study lead to the conclusion that the SDT process model for exercise behaviour can be extended by considering an additional connection between exercise causality orientations and exercise participation goals (Fig 1; dashed line). This relationship emphasizes the importance to consider individual differences in understanding the process of motivated exercise behaviour. Specifically, exercise causality orientations seem to influence exercise specific motivation, which can, overall, lead to more sustainable physical activity and overall health [7,8].

Beyond the background of the SDT process model for exercise behaviour, it can be assumed that interindividual differences in assumptions about the initiation of behaviour can lead to different levels of basic needs satisfaction. However, it stays unclear how the interindividual differences can also influence the evaluation and perception of contextual factors or the specific goal setting. In this context, potential interaction effects between exercise causality orientations and exercise participation goals/contextual factors on exercise basic needs satisfaction should be addressed in future studies. Also, it stays unclear if and how exercise causality orientations do not only interrelate, but also interact with exercise participation goals and situational components, leading to different degrees of exercise basic needs satisfaction and thus intrinsically motivated behaviour. In this context, the interindividual differences in causality orientations could also potentially influence the evaluation and perception of contextual factors. Specifically, individual differences in exercise causality orientations might potentially lead to specific exercise goal setting, or might guide the interpretation of situations associated with specific goals. To answer these questions, further longitudinal studies examining these SDT related constructs are needed.

## General discussion and conclusions

The main aim of this work was to translate and validate the exercise causality orientations scale ECOS [20] to German. The ECOS is based on the general causality orientations scale GCOS [29] and is rooted in the SDT [5,6]. The SDT is a well-established and widely used theory explaining motivated behaviour in manifold contexts and that contributes to understand motivated behaviour, such as health and exercise behaviour [9,12,58,59]. In this context, the assessment of exercise causality orientations can contribute to a better understanding how situations are interpreted as informational, controlling, or amotivating, promoting intrinsic vs. extrinsic forms of motivation. As a consequence, the prevalence of a full set of assessment tools capturing aspects of the SDT can be crucial precise predictions of motivated health and exercise behaviour. In this work, the G-ECOS was successfully translated, validated, and cross-validated via three studies and independent samples. The analyses were guided by the methodology applied in original ECOS validation study, and the guidelines for high quality and valuable translation and validation of questionnaires [54]. Also, it was a pronounced aim of this work to provide transparency throughout all analyses of this work and to make this research verifiable and reproducible. In doing so, open data, code, and material was made accessible. Furthermore, raw data of the original ECOS questionnaire was obtained to provide comparisons with the original validation study. Compared to the ECOS, both studies used a nine-vignette version of the questionnaire as a starting point of item selection. However, the analyses in this work yielded an item selection of only four vignettes (compared to the ECOS comprising 7 vignettes). Thus, the G-ECOS represents a valid, but short vignette-based questionnaire that therefore displays limitations in reliability coefficients. Consequently, this scale should be used with caution for *individual* assessment until evidence for its reliability and validity beyond group-level research has been obtained. Hence, future studies are required to test the questionnaire in individual settings. Nevertheless, the G-ECOS represents a useful tool for researchers and practitioners to elaborate on the individual differences that might substantially influence the motivational sequence of persons. Based on this knowledge, it should also be investigated how differences in exercise causality orientations can be considered to promote specific exercise participation goals or situational features in exercisers with the aim to ensure maintained and motivated health and exercise behavior.

In this context, the results of Study 3 can serve to gain a first insight into the process and antecedents of motivated health behaviour. Based on these cross-sectional results, further longitudinal studies are needed to elaborate on the *causal* processes and interactions between exercise causality orientations, situational factors, and exercise participation goals on the sequence of motivated health and exercise behaviour.

In sum, the German G-ECOS was translated and validated throughout three studies included in this work. Furthermore, the results of this work can contribute to theory building within the SDT process model for exercise behaviour [8], indicating that the model can be advanced by adding an additional connection between exercise causality orientations and exercise participation goals.

All authors of this manuscript state that there is no financial interest or benefit arising from the direct applications of this research.

## Supporting information

S1 Table Final items of the G-ECOS. The final G-ECOS includes the vignettes 4, 6, 8, and 9 (thus entailing 12 items). Each item is rated on a seven-point Likert scale (1 = very unlikely to 7 = very likely).
(DOCX)

S2 Table Results of the initial translation and back-translation including indication of changes. (DOCX)

S3 Table Model fit indices and model comparison for the CTCU and CT models. CTCU, correlated traits/correlated uniqueness; CT, correlated traits.
(DOCX)

S4 Table Bayesian pearson correlations with related constructs (*n* = 548). * BF_10_ > 10, **, BF_10_ > 30, *** BF_10_ > 100.
(PDF)
