# Supplementary material for: Validation of the vignette-based German Exercise Causality Orientation Scale (G-ECOS)
Source: PLoS One. 2019 Oct 10;14(10):e0223643. doi: 10.1371/journal.pone.0223643 (PMC6786641; doi:10.1371/journal.pone.0223643)
Supplement: S1 Table — The final G-ECOS includes the vignettes 4, 6, 8, and 9 (thus entailing 12 items). Each item is rated on a seven-point Likert scale (1 = very unlikely to 7 = very likely). (DOCX) [file pone.0223643.s001.docx]

S1 Table. Final Items of the G-ECOS

| **Scale (Code)** | **German G-ECOS vignette** | **Original ECOS vignette** |
| --- | --- | --- |
| **Vignette 1** |  |  |
|  | Sie beginnen ein neues Trainingsprogramm. Wahrscheinlich… | You are beginning a new exercise programme. You are likely to: |
| 1. Control (Contr1a) | besuchen Sie einen strukturierten Trainingskurs, in dem ein Kursleiter/ eine Kursleiterin Ihnen sagt, was zu tun ist | Attend a structured exercise class where an exercise leader is telling you what to do. |
| 1. Autonomy (Auto1b) | besuchen Sie ein Fitnessstudio, wo Sie für sich selbst entscheiden, welche Übungen Sie durchführen. | Attend a gym where you decide for yourself which exercise to complete. |
| 1. Impersonal (Imp1c) | gehen Sie mit ihren Freunden/ Freundinnen mit und tun, was Ihre Freunde/ Freundinnen tun. | Tag along with your friends and do what they do. |
| **Vignette 2** |  |  |
|  | Sie beginnen ein neues Trainingsprogramm. Wahrscheinlich… | You are beginning a new exercise programme. You are likely to: |
| 1. Autonomy (Auto2a) | sind Sie interessiert an der neuen Herausforderung und freuen sich darauf, Nutzen davon zu ziehen. | Feel interested in the new challenge and look forward to feeling its benefits. |
| 1. Control (Contr2b) | freuen Sie sich, Gewicht zu verlieren, Ihr Aussehen zu verbessern, Ihre Fitness zu verbessern, etc. | Look forward to losing weight, improving your appearance, increasing your fitness, etc. |
| 1. Impersonal (Imp2c) | fühlen Sie sich gestresst und ängstlich über die neue Situation. | Feel stressed and anxious about the new situation. |
| **Vignette 3** |  |  |
|  | Sie werden gebeten, all ihr wöchentliches Training in einem Trainingstagebuch festzuhalten. Wahrscheinlich sehen Sie das Trainingstagebuch… | You are asked to keep a record of all the weekly exercise you have completed in an exercise diary. You are likely to view the diary: |
| 1. Impersonal (Imp3a) | als eine Erinnerung, wie unfähig Sie sind, diese Aufgabe auszufüllen. | As a reminder of how incapable you are at fulfilling the task. |
| 1. Autonomy (Auto3b) | als einen Weg, Ihren Fortschritt zu messen und stolz auf Ihre Leistung zu sein. | As a way to measure your progress and to feel proud of your achievements. |
| 1. Control (Contr3c) | als einen weg, sich zum Training zu zwingen. | As a way of pressurizing yourself to exercise. |
| **Vignette 4** |  |  |
|  | Um zu überwachen, wie gut Sie in einem Trainingsprogramm abschneiden, wollen Sie wahrscheinlich... | To monitor how well you are doing in an exercise programme, you are likely to want: |
| a. Control (Contr4a) | eine Menge Lob und Ermutigung von anderen bekommen. | To be given a lot of praise and encouragement from others. |
| b. Autonomy (Auto4b) | Ihre Leistung selbst bewerten und sich positives Feedback geben. | To evaluate your own performance and provide yourself with positive feedback. |
| c. Impersonal (Imp4c) | einfach nur hoffen, dass alles was Sie tun, korrekt ist. | To just hope that what you are doing is correct. |
| **Vignette 5** |  |  |
|  | In den letzten 6 Monaten haben Sie regelmäßig trainiert, aber in letzter Zeit haben Sie Trainingseinheiten verpasst du finden es schwierig, sich zum Training zu motivieren. Wahrscheinlich… | You have been exercising regularly for 6 months but recently you have been missing sessions and are finding it hard to get motivated to exercise. You are likely to: |
| 1. Control (Contr5a) | richten Sie sich an jemanden, der hilft Sie zu motivieren. | Approach someone to help motivate you. |
| 1. Impersonal (Imp5b) | ignorieren Sie das Problem, nichts kann getan werden um Ihre Motivation zu verbessern | Ignore the problem, nothing can be done to improve your motivation. |
| 1. Autonomy (Auto5c) | nutzen Sie Ihre eigenen Strategien, um sich selbst zu motivieren. | Employ your own strategies to motivate yourself. |
| **Vignette 6** |  |  |
|  | Wenn Sie einen Fitnesstrainer/eine Fitnesstrainerin hätten, der/die Ihnen ein Trainingsprogramm zum Befolgen geben würde, würden Sie wahrscheinlich... | If you were going to see a fitness instructor to get an exercise programme to follow, you would likely: |
| a. Autonomy (Auto6a) | wollen, in die Entscheidung einbezogen zu werden, was in das Programm hereinkommt. | Want to be involved in making decisions about what goes in the programme. |
| b. Impersonal (Imp6b) | wollen, dass das Programm so ausgedacht wird, wie es schon in der Vergangenheit getan wurde. | Want the programme to be devised the way it had been done in the past. |
| c. Control (Contr6c) | wollen, den Fitnesstrainer/die Fitnesstrainerin entscheiden zu lassen welches Training Sie ausführen. | Want to let the fitness instructor decide what exercises you should do. |
| **Vignette 7** |  |  |
|  | Ihnen wurde gesagt, dass sich Ziele zu setzen ein guter Weg ist, um sich zum Training zu motivieren. Sie würden wahrscheinlich… | You have been told that setting goals is a good way to motivate yourself to exercise. You would likely: |
| 1. Autonomy (Auto7a) | sich eigene realistische, aber anspruchsvolle Ziele setzen. | Set your own realistic but challenging goals. |
| 1. Control (Contr7b) | jemanden, der/ die Ihnen wichtig ist dazu bringen, die Ziele für Sie zu setzen. | Make someone important to you set goals for you to aim. |
| 1. Impersonal (Imp7c) | sich keine Ziele setzen, weil Sie diese vielleicht nicht umsetzen könnten. | Not set goals because you may not be able to live up to them. |
| **Vignette 8** |  |  |
|  | Während einer Diskussion mit einem Trainingsberater/einer Trainingsberaterin präsentiert er/sie viele Optionen für den besten Weg, wie Sie trainieren und Fitness und gesundheitlichen Nutzen erreichen können. Wahrscheinlich ist Ihr erster Gedanke... | During a discussion with an exercise counsellor, he or she presents many options on the best way for you to exercise to achieve fitness and health benefits. It is likely that your first thought would be: |
| a. Control (Contr8a) | Was denken Sie (der Trainingsberater/die Trainingsberaterin), soll ich tun? | What do you (the exercise leader) think I should do? |
| b. Autonomy (Auto8b) | Was denke ich, ist die beste Option für mich? | What do I think is the best option for me? |
| c. Impersonal (Imp8c) | Was haben alle anderen in der Vergangenheit getan? | What has everyone else done in the past? |
| **Vignette 9** |  |  |
|  | Wie hart Sie während eines Trainings trainieren, ist wahrscheinlich abhängig von... | During an exercise session, how hard you are working out is likely to be governed by: |
| a. Control (Contr9a) | der Trainingsintensität, die Ihnen aufgetragen wurde. | The intensity you have been told to exercise at. |
| b. Impersonal (Imp9b) | dem, was alle anderen um Sie herum tun. | What everyone around you is doing. |
| c. Autonomy (Auto9c) | dem, wie Sie sich fühlen, wenn Sie mit der von Ihnen gewählten Intensität trainieren. | How you are feeling while exercising at the intensity you choose. |

The final G-ECOS includes the vignettes 4, 6, 8, and 9 (thus entailing 12 items). Each item is rated on a seven-point Likert scale (1 = very unlikely to 7 = very likely).
