# Supplementary material for: Validation of the vignette-based German Exercise Causality Orientation Scale (G-ECOS)
Source: PLoS One. 2019 Oct 10;14(10):e0223643. doi: 10.1371/journal.pone.0223643 (PMC6786641; doi:10.1371/journal.pone.0223643)
Supplement: S2 Table — (DOCX) [file pone.0223643.s002.docx]

**S2 Table.** **Results of the Initial Translation and Back-Translation Including Indication of Changes.**

| **Vignette No.** | **Initial translation** | **Back translation** | **Changes in final version** | **Used in** |
| --- | --- | --- | --- | --- |
| 1 | Sie beginnen ein neues Trainingsprogramm. Wahrscheinlich...  a. besuchen Sie einen strukturierten **Trainingskurs**, **in dem** ein Kursleiter/ eine Kursleiterin Ihnen sagt, was zu tun ist.  b. besuchen Sie ein Fitnessstudio, wo Sie für sich selbst entscheiden, welche **Übungen** Sie durchführen.  c. gehen Sie mit Ihren Freunden/ Freundinnen mit und tun, was Ihre Freunde/ Freundinnen tun. | You are starting a new exercise program. You are probably …  a. attending a structured course, in which an instructor tells you what to do.  b. going to a gym, where you can decide which training program you will do yourself.  c. tagging along with your friends and doing what they do. | Kurs, wo  welches Training | ECOS only |
| 2 | Sie beginnen ein neues Trainingsprogramm. Wahrscheinlich...  a. sind Sie interessiert an der neuen Herausforderung und freuen sich darauf, Nutzen davon zu ziehen.  b. freuen Sie sich, Gewicht zu verlieren, Ihr Aussehen zu verbessern, Ihre Fitness zu verbessern, etc.  c. fühlen Sie sich gestresst und ängstlich über die neue Situation. | You are starting a new exercise program. You are probably …  a. interested in the new challenge and excited about its benefits.  b. looking forward to losing weight, improving your look, your fitness etc.  c. feeling stressed and worried about the news situation. |  | none |
| 3 | Sie werden gebeten, all ihr wöchentliches Training in einem Trainingstagebuch festzuhalten. Wahrscheinlich sehen Sie das Trainingstagebuch...  a. als eine Erinnerung, wie unfähig Sie sind, diese Aufgabe auszufüllen.  b. als einen Weg, Ihren Fortschritt zu messen und stolz auf Ihre Leistung zu sein.  c. als einen Weg, sich zum Training zu zwingen. | You are asked to document your weekly exercises in an exercise diary. You probably consider the exercise diary to be …  a. a reminder of your inability to complete this exercise.  b. a way to measure your progress and be proud of your achievement.  c. a way to force yourself to do the exercises. |  | ECOS only |
| 4 | Um zu überwachen, wie gut Sie in einem Trainingsprogramm abschneiden, wollen Sie wahrscheinlich...  a. eine Menge Lob und Ermutigung von anderen bekommen.  b. ihre Leistung selbst bewerten und sich positives Feedback geben.  c. einfach nur hoffen, dass alles was Sie tun, korrekt ist. | In order to monitor how well you perform in an exercise program, you probably …  a. want to receive a lot of praise and encouragement from others.  b. want to evaluate your own performance and give yourself positive feedback.  c. simply hope that everything you are doing is correct. |  | ECOS &  G-ECOS |
| 5 | In den letzten 6 Monaten haben Sie regemäßig trainiert, aber in letzter Zeit haben Sie Trainingseinheiten **verpasst** und finden es schwierig, sich zum Training zu motivieren. Wahrscheinlich...  a. richten Sie sich an jemanden, der hilft Sie zu motivieren.  b. ignorieren Sie das Problem, nichts kann getan werden um Ihre Motivation zu verbessern.  c. nutzen Sie Ihre eigenen Strategien, um sich selbst zu motivieren. | You have exercised regularly over the past six months but lately you have skipped workouts and find it difficult to motivate yourself to exercise. You probably …  a. turn to someone to help you get motivated.  b. ignore the problem, nothing can be done to improve your motivation.  c. use your own strategies to motivate yourself. | ausgelassen | ECOS only |
| 6 | Wenn Sie einen Fitnesstrainer/ eine Fitnesstrainerin hätten, der/ die Ihnen ein Trainingsprogramm zum Befolgen geben würde, würden Sie wahrscheinlich...  a. wollen, in die Entscheidung einbezogen zu werden, was in das Programm hereinkommt.  b. wollen, dass das Programm so ausgedacht wird, wie es schon in der Vergangenheit getan wurde.  c. wollen, den Fitnesstrainer/ die Fitnesstrainerin entscheiden zu lassen welches Training Sie ausführen. | If you had a fitness coach who gave you an exercise program to follow, you would probably …  a. want to be part of the decision process as to what is included in the program.  b. want the program to be designed the same way as in the past.  c. want the fitness coach to decide which exercises to do. |  | G-ECOS only |
| 7 | Ihnen wurde gesagt, **dass sich Ziele zu setzen ein guter Weg ist**, um sich zum Training zu motivieren. Sie würden wahrscheinlich...  a. sich eigene realistische, aber anspruchsvolle Ziele setzen.  b. jemanden, der/ die Ihnen wichtig is**t** **dazu bringen, die Ziele für Sie zu setzen.**  c. sich keine Ziele setzen, weil Sie diese vielleicht nicht umsetzen könnten. | You were told that a good way to motivate yourself for workouts is to set yourself goals. You probably would …  a. set your own realistic but challenging goals.  b. let someone who is important to you set your goals for you.  c. not set any goals because you might not be able to reach them. | Dass es ein guter Weg ist, sich Ziele zu setzen  wichtig ist, die Ziele für Sie setzen lassen | ECOS only |
| 8 | Während einer Diskussion mit einem Trainingsberater/ einer Trainingsberaterin präsentiert er/ sie viele Optionen für den besten Weg, wie sie trainieren und Fitness und gesundheitlichen Nutzen erreichen können. Wahrscheinlich ist ihr erster Gedanke...  a. Was denken Sie (der Trainingsberater/ die Trainingsberaterin), soll ich tun?  b. Was denke ich, ist die beste Option für mich?  c. Was haben alle anderen in der Vergangenheit getan? | During a discussion with an exercise advisor, he presents you with many options for the best way to exercise and achieve fitness and health benefits. Your first thought is probably …  a. What do you (the exercise advisor) think I should do?  b. What do I think is the best option for me?  c. What did everyone else in the past do? |  | ECOS &  G-ECOS |
| 9 | Wie hart sie während eines Trainings trainieren, ist wahrscheinlich abhängig von...  a. der Trainingsintensität, die Ihnen aufgetragen wurde.  b. dem, was alle anderen um Sie herum tun.  c. dem, wie Sie sich fühlen, wenn Sie mit der von Ihnen gewählten Intensität trainieren. | How hard you exercise during a workout probably depends on …  a. the workout intensity you were tasked with.  b. what everyone else around you is doing.  c. how you feel while exercising with the intensity you chose. |  | ECOS &  G-ECOS |

The original ECOS items and the final G-ECOS items are provided in S1 Table.
