# Supplementary material for: Validation of the vignette-based German Exercise Causality Orientation Scale (G-ECOS)
Source: PLoS One. 2019 Oct 10;14(10):e0223643. doi: 10.1371/journal.pone.0223643 (PMC6786641; doi:10.1371/journal.pone.0223643)
Supplement: S3 Table — CTCU, correlated traits/correlated uniqueness; CT, correlated traits. (DOCX) [file pone.0223643.s003.docx]

| **Model** | **Scaled** $\boldsymbol{\chi}^{\boldsymbol{2}}$ | ***df*** | ***p*** | **CFI** | **TLI** | **SRMR** | **RMSEA** | **90%-CI for RMSEA** | $\boldsymbol{\Delta}\boldsymbol{\chi}^{\boldsymbol{2}}$**of comparison with previous model** | $\boldsymbol{\Delta}\boldsymbol{\chi}^{\boldsymbol{2}}$***df*** | $\boldsymbol{\Delta}\boldsymbol{\chi}^{\boldsymbol{2}}$***p*** |
| --- | --- | --- | --- | --- | --- | --- | --- | --- | --- | --- | --- |
| **9 vignettes** |  |  |  |  |  |  |  |  |  |  |  |
| CTCU | 524.05 | 269 | <.001 | .81 | .77 | .09 | .06 | .05; .07 |  |  |  |
| CT | 793.27 | 269 | <.001 | .65 | .61 | .10 | .08 | .07; .08 | 186.55 | 27 | < .001 |
| **Final 4 vignettes** |  |  |  |  |  |  |  |  |  |  |  |
| CTCU | 54.05 | 39 | .055 | .96 | .93 | .05 | .04 | .00; .06 |  |  |  |
| CT | 407.21 | 39 | <.001 | .75 | .66 | .07 | .09 | .07; .10 | 78.22 | 12 | < .001 |

**S3 Table. Model Fit Indices and Model Comparison for the CTCU and CT Models.**

CTCU, correlated traits/correlated uniqueness; CT, correlated traits.
