# Supplementary material for: Validation of the vignette-based German Exercise Causality Orientation Scale (G-ECOS)
Source: PLoS One. 2019 Oct 10;14(10):e0223643. doi: 10.1371/journal.pone.0223643 (PMC6786641; doi:10.1371/journal.pone.0223643)
Supplement: S4 Table — * BF10 > 10, **, BF10 > 30, *** BF10 > 100. (PDF) [file pone.0223643.s004.pdf]

	Autonomy Orientation		Control Orientation		Impersonal Orientation		Intrinsic Regulation		Identified Regulation		Introjected Regulation		Extrinsic Regulation		Autonomy Need Satisfaction		Competence Need Satisfaction		Relatedness Need Satisfaction		Fitness/ Health Goal		Appearance/ Physique Goal		Distraction/ Catharsis Goal		Activation/ Pleasure Goal		Aesthetics Goal		Competition/ Achievement Goal		Contact Goal	
	r (95% CI)	BF10	r (95% CI)	BF10	r (95% CI)	BF10	r (95% CI)	BF10	r (95% CI)	BF10	r (95% CI)	BF10	r (95% CI)	BF10	r (95% CI)	BF10	r (95% CI)	BF10	r (95% CI)	BF10	r (95% CI)	BF10	r (95% CI)	BF10	r (95% CI)	BF10	r (95% CI)	BF10	r (95% CI)	BF10	r (95% CI)	BF10	r (95% CI)	BF10
Autonomy Orientation	-	-	-	-	-	-	-	-	-	-	-	-	-	-	-	-	-	-	-	-	-	-	-	-	-	-	-	-	-	-	-	-	-	
Control Orientation	0.025 (0.055; 0.104)	0.061	-	-	-	-	-	-	-	-	-	-	-	-	-	-	-	-	-	-	-	-	-	-	-	-	-	-	-	-	-	-	-	
Impersonal Orientation	0.027 (0.106; 0.052)	0.063	0.395 (0.324; 0.459)	7.981e+20	-	-	-	-	-	-	-	-	-	-	-	-	-	-	-	-	-	-	-	-	-	-	-	-	-	-	-	-	-	
Intrinsic Regulation	0.293*** (0.217;0.364)	1.372e+10	-0.034 (0.114; 0.047)	0.072	-0.130 (-0.208; -0.050)	7.696	-	-	-	-	-	-	-	-	-	-	-	-	-	-	-	-	-	-	-	-	-	-	-	-	-	-	-	
Identified Regulation	0.339*** (0.265;0.408)	1.918e+14	0.085 (0.004; 0.164)	0.436	-0.044 (-0.124; 0.037)	0.090	0.591*** (0.536;0.639)	7.732e+56	-	-	-	-	-	-	-	-	-	-	-	-	-	-	-	-	-	-	-	-	-	-	-	-	-	
Introjected Regulation	0.034 (-0.047; 0.114)	0.072	0.294*** (0.218;0.365)	1.819e+10	0.230*** (0.152;0.305)	455719.860	0.019 (-0.059; 0.097)	0.056	0.275*** (0.200; 0.345)	2.169e+9	-	-	-	-	-	-	-	-	-	-	-	-	-	-	-	-	-	-	-	-	-	-	-	
Extrinsic Regulation	-0.144* (-0.221; -0.064)	23.552	0.211*** (0.132;0.286)	31187.667	0.240*** (0.162; 0.314)	1.895e+6	-0.185*** (-0.259; -0.108)	2532.483	-0.103 (-0.179; -0.025)	1.370	0.306*** (0.233; 0.374)	1.030e+12	-	-	-	-	-	-	-	-	-	-	-	-	-	-	-	-	-	-	-	-	-	
Autonomy Need Satisfaction	0.271*** (0.194; 0.343)	2.726e+8	-0.060 (-0.140; 0.021)	0.150	-0.100 (-0.179; -0.019)	0.985	0.599*** (0.544; 0.647)	5.980e+55	0.482*** (0.417; 0.540)	4.335e+32	0.028 (-0.053; 0.107)	0.064	-0.177*** (-0.254; -0.098)	650.347	-	-	-	-	-	-	-	-	-	-	-	-	-	-	-	-	-	-	-	
Competence Need Satisfaction	0.288*** (0.212; 0.360)	5.822e+9	-0.017 (-0.097; 0.064)	0.056	-0.097 (-0.176; -0.016)	0.812	0.632*** (0.580; 0.677)	1.186e+64	0.517*** (0.455; 0.573)	5.108e+38	-0.013 (-0.093: 0.067)	0.054	-0.258*** (-0.331; -0.181)	3.852e+7	0.737*** (0.689; 0.770)	3.512e+104	-	-	-	-	-	-	-	-	-	-	-	-	-	-	-	-	-	
Relatedness Need Satisfaction	0.137* (0.057;0.215)	13.525	0.057 (-0.024; 0.137)	0.134	0.032 (-0.049; 0.112)	0.069	0.520*** (0.458; 0.576)	1.743e+39	0.319*** (0.245; 0.389)	3.314e+12	0.018 (-0.062; 0.098)	0.057	-0.020 (-0.100; 0.060)	0.058	0.495*** (0.433; 0.551)	5.776e +36	0.505*** (0.443; 0.560)	3.450e+38	-	-	-	-	-	-	-	-	-	-	-	-	-	-	-	
Fitness/ Health Goal	0.203*** (0.124; 0.279)	10847.240	0.123 (0.043; 0.202)	4.449	0.025 (-0.056; 0.106)	0.062	0.235*** (0.157;0.309)	913451.891	0.482*** (0.417; 0.541)	3.318e+32	0.224*** (0.146; 0.299)	199681.688	-0.029 (-0.109; 0.051)	0.066	0.223*** (0.145; 0.297)	171730.705	0.233*** (0.155; 0.307)	777457.918	0.128 (0.048; 0.206)	6.979	-	-	-	-	-	-	-	-	-	-	-	-	-	
Appearance/ Physique Goal	-0.024 (-0.105; 0.057)	0.061	0.183*** (0.103; 0.259)	974.023	0.112 (0.031; 0.191)	2.034	-0.045 (-0.125; 0.036)	0.093	0.202*** (0.123; 0.277)	10816.272	0.296*** (0.220; 0.367)	2.698e+10	0.049 (-0.032; 0.128)	0.103	-0.025 (-0.105; 0.056)	0.062	-0.023 (-0.103; 0.057)	0.060	-0.042 (-0.121; 0.039)	0.086	0.337*** (0.264; 0.405)	4.321e+14	-	-	-	-	-	-	-	-	-	-	-	
Distraction/ Catharsis Goal	0.121 (0.040; 0.199)	3.641	0.136* (0.055; 0.214)	11.191	0.112 (0.031; 0.191)	1.974	0.393*** (0.322; 0.458)	1.311e+20	0.312*** (0.237; 0.382)	6.494e+11	0.111 (0.031; 0.190)	2.015	-0.025 (-0.105; 0.056)	0.062	0.242*** (0.164; 0.315)	2.594e +6	0.250*** (0.173; 0.323)	1.003e +7	0.216*** (0.138; 0.291)	70747.065	0.238*** (0.161; 0.311)	2.530e +6	0.200*** (0.123; 0.275)	12940.099	-	-	-	-	-	-	-	-	-	
Activation/ Pleasure Goal	0.254*** (0.176; 0.327)	1.234e+7	0.003 (-0.078; 0.084)	0.052	-0.085 (-0.165; -0.004)	0.428	0.619*** (0.566; 0.666)	3.192e+60	0.442*** (0.374; 0.504)	2.971e+26	-0.050 (-0.129; 0.031)	0.106	-0.217*** (-0.292; -0.138)	73143.915	0.421*** (0.351; 0.484)	4.707e +23	0.445*** (0.377; 0.506)	9.218e+26	0.404*** (0.334; 0.469)	3.857e+21	0.361*** (0.290; 0.428)	1.550e+17	0.007 (-0.072; 0.086)	0.051	0.568*** (0.511; 0.618)	1.336e+50	-	-	-	-	-	-	-	
Aesthetics Goal	0.202*** (0.122; 0.277)	8675.285	-0.004 (-0.085; 0.077)	0.052	-0.010 (-0.090; 0.071)	0.053	0.444*** (0.376; 0.506)	6.494e+26	0.284*** (0.207; 0.356)	2.743e+9	0.008 (-0.073; 0.088)	0.052	-0.076 (-0.156; 0.004)	0.287	0.284*** (0.208; 0.356)	3.216e +9	0.295*** (0.220; 0.366)	2.546e+10	0.295*** (0.220; 0.366)	2.531e+10	0.170*** (0.091; 0.245)	366.496	-0.046 (-0.125; 0.033)	0.097	0.269*** (0.193; 0.340)	4.131e +8	0.459*** (0.393; 0.518)	7.583e+29	-	-	-	-	-	
Competition/Achievement Goal	0.127 (0.047; 0.206)	5.925	0.099 (0.018; 0.178)	0.890	0.016 (-0.065; 0.096)	0.056	0.500*** (0.436; 0.557)	2.761e+35	0.270*** (0.193; 0.342)	2.221e+8	0.068 (-0.012; 0.148)	0.204	0.043 (-0.037; 0.123)	0.089	0.326*** (0.251; 0.395)	1.222e +13	0.378*** (0.306; 0.444)	3.178e+18	0.433*** (0.365; 0.496)	2.306e+25	0.061 (-0.018; 0.139)	0.156	-0.050 (-0.129; 0.029)	0.110	0.285*** (0.210; 0.356)	8.054e +9	0.345*** (0.273; 0.413)	3.054e+15	0.389*** (0.319; 0.453)	2.331e+20	-	-	-	
Contact Goal	-0.001 (-0.082; 0.079)	0.052	0.073 (-0.008; 0.153)	0.243	0.110 (0.029; 0.189)	1.821	0.379*** (0.307; 0.445)	2.968e+18	0.103 (0.023; 0.182)	1.216	0.008 (-0.072; 0.089)	0.053	0.167*** (0.087; 0.244)	209.056	0.150** (0.070; 0.227)	41.082	0.168*** (0.088; 0.244)	228.238	0.582*** (0.525; 0.632)	4.805e+51	0.013 (-0.066; 0.092)	0.053	-0.008 (-0.087; 0.071)	0.052	0.233*** (0.156; 0.306)	1.155e +6	0.327*** (0.254; 0.395)	4.367e+13	0.270*** (0.194; 0.341)	4.743e +8	0.500*** (0.437; 0.556)	4.617e+36	-	
